# *N*-Cyclo­hexyl-3-methyl­benzamidine

**DOI:** 10.1107/S1600536813006272

**Published:** 2013-03-09

**Authors:** Rui-Qin Liu, Sheng-Di Bai, Tao Wang

**Affiliations:** a Institute of Applied Chemistry, Shanxi University, Taiyuan 030006, People’s Republic of China

## Abstract

The title amidine compound, C_14_H_20_N_2_, prepared by a one-pot reaction, is asymmetric as only one N atom has an alkyl substituent. The terminal cyclo­hexyl group connected to the amino N atom is located on the other side of the N—C—N skeleton to the 4-methylbenzene ring and has a chair conformation. The dihedral angle between the phenyl ring and the NCN plane is 47.87 (12)°. In the crystal, mol­ecules are linked *via* N—H⋯N hydrogen bonds, forming chains propagating along the *a*-axis direction.

## Related literature

For reviews of related metal amidinates and their applications in olefin polymerization, see: Edelmann (1994 ▶); Barker & Kilner (1994 ▶); Collins (2011 ▶); Bai *et al.* (2010 ▶); Yang *et al.* (2013 ▶). For a review of neutral amidines, see: Coles (2006 ▶). For a related synthetic method for amidines, see: Wang *et al.* (2008 ▶). For related silyl-linked bis­(amidinate) ligands, see: Bai *et al.* (2006 ▶).
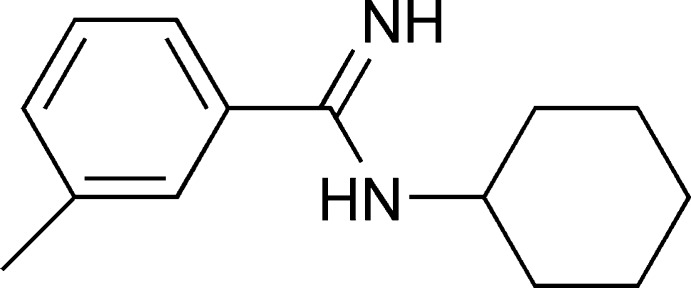


## Experimental

### 

#### Crystal data

C_14_H_20_N_2_
*M* *_r_* = 216.32
Orthorhombic, 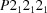

*a* = 9.064 (2) Å
*b* = 11.417 (3) Å
*c* = 12.311 (3) Å
*V* = 1274.0 (5) Å^3^
*Z* = 4
Mo *K*α radiation
μ = 0.07 mm^−1^
*T* = 200 K
0.30 × 0.25 × 0.20 mm

#### Data collection

Bruker SMART CCD diffractometer
Absorption correction: multi-scan (*SADABS*; Sheldrick, 1996 ▶) *T*_min_ = 0.980, *T*_max_ = 0.987
7147 measured reflections
2244 independent reflections
1758 reflections with *I* > 2σ(*I*)
*R*_int_ = 0.042

#### Refinement

*R*[*F* ^2^ > 2σ(*F* ^2^)] = 0.040
*wR*(*F* ^2^) = 0.098
*S* = 1.02
2244 reflections
154 parameters
H atoms treated by a mixture of independent and constrained refinement
Δρ_max_ = 0.15 e Å^−3^
Δρ_min_ = −0.12 e Å^−3^

### 

Data collection: *SMART* (Bruker, 2000 ▶); cell refinement: *SAINT* (Bruker, 2000 ▶); data reduction: *SAINT*; program(s) used to solve structure: *SHELXS97* (Sheldrick, 2008 ▶); program(s) used to refine structure: *SHELXL97* (Sheldrick, 2008 ▶); molecular graphics: *SHELXTL* (Sheldrick, 2008 ▶); software used to prepare material for publication: *SHELXL97*.

## Supplementary Material

Crystal structure: contains datablock(s) I, global. DOI: 10.1107/S1600536813006272/rk2395sup1.cif

Structure factors: contains datablock(s) I. DOI: 10.1107/S1600536813006272/rk2395Isup2.hkl

Supplementary material file. DOI: 10.1107/S1600536813006272/rk2395Isup3.cml

Additional supplementary materials:  crystallographic information; 3D view; checkCIF report

## Figures and Tables

**Table 1:** Hydrogen-bond geometry (Å, °)

*D*—H⋯*A*	*D*—H	H⋯*A*	*D*⋯*A*	*D*—H⋯*A*
N1—H1*A*⋯N2^i^	0.90 (2)	2.08 (2)	2.975 (2)	168.0 (18)

Symmetry code: (i) 
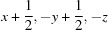
.

**Fig. 1.**
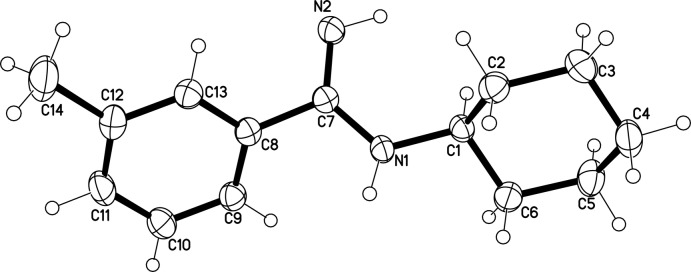
The molecular structure, showing the atom–numbering scheme. Displacement ellipsoids are drawn at the 30% probability level. H atoms are presented as a small spheres of arbitrary radius.

**Fig. 2.**
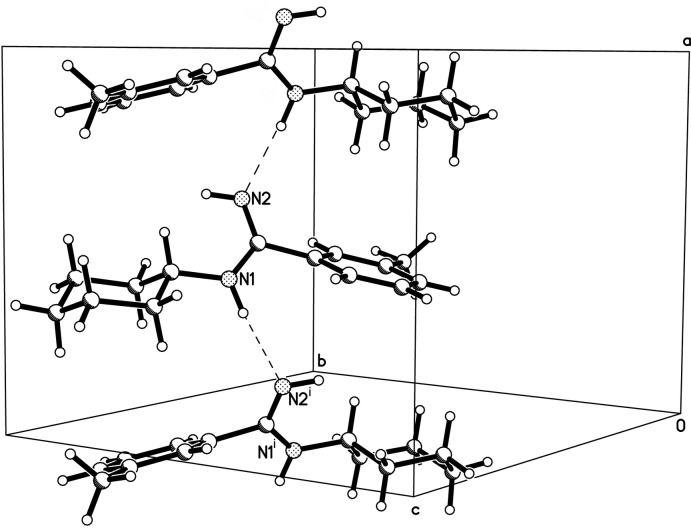
The view of one–dimensional chain in crystal structure of I. Symmetry codes: (i) x+1/2, -y+1/2, -z.

## supplementary crystallographic information

### Comment

The exploration of ancillary ligand systems supporting catalytically active metal centers is a long–standing demand in the coordination chemistry. Amidinates represent an important class in the array comparable to the cyclopentadienyl system (Edelmann, 1994; Barker & Kilner, 1994; Collins, 2011). They are four–electron, monoanionic and *N*–donor bidentate chelates, demonstrating a great diversity by variation of substituents on the conjugated N—C—N backbone. Their steric and electronic properties are easily tunable to meet therequirements of different metal centers. In the course of extending amidinate chemistry, we explored a synthetic pathway to the silyl–linked bis(amidinate) ligands, [Si*Me*_2_{NC(*Ph*)N(*R*)}_2_]^2-^ (Bai *et al.*, 2006). They were applied to synthesize the Group 4 complexes, which were good catalysts for ethylene polymerization (Bai *et al.*, 2010; Yang *et al.*, 2013). Amidines are convenient precursors for both monoanionic amidinate ligands and bianionic *ansa*–bis(amidinate) ligands (Coles, 2006). Some amidines could be prepared by Yb complex catalyzed addtion reactions of aromatic amines and nitriles (Wang *et al.*, 2008). Here, the synthesis and crystal structure of a new amidine will be described.

The title compound I was prepared by a one–pot reaction of cyclohexylamine, Li*Bu*, *m*–tolunitrile and H_2_O. The intermediate process involved an addition reaction of lithium amide and nitrile to yield lithium monoamidinate. The suitable for X–ray investigation single–crystal of the title compound was obtained by recrystallization in CH_2_Cl_2_. Its molecular structure is shown in Fig. 1. Two N atoms connect the central C atom in different lengths of 1.357 (2)Å and 1.284 (2)Å, composing the characteristic N—C—N skeleton for amidine species. The terminal cyclohexyl with chair–like configuration connects the amino N atom. The phenyl group is attached to the central C atom. The angle between the phenyl plane and the [NCN] plane is 47.87 (12)°. Cyclohexyl and phenyl are in opposite directions. Fig. 2 displays the packing view of compound I. Molecules of I can form the one–dimensional chain extending along *a*–axis through the intermolecular hydrogen bond. The imino N atom is the acceptor for hydrogen atom. In the chain, every adjacent two molecules have *C_2_* rotation symmetrical relationship with each other and the couple serves as the repeatable unit.

### Experimental

A solution of Li*Bu* (2.2 *M*, 2.7 ml, 6.0 mmol) in hexane was slowly added into a stirred solution of cyclohexylamine (0.69 ml, 6.0 mmol) in *Et*_2_O (*ca* 30 ml) by syringe at 273 K. The reaction mixture was warmed to room temperature and kept stirring for 3 h. Then *m*–tolunitrile (0.71 ml, 6.0 mmol) was added by syringe at 273 K. The reaction mixture was warmed to room temperature and kept stirring for 4 h. H_2_O (0.11 ml, 6.0 mmol) was added by syringe at 273 K. After stirred at room temperature for 4 h, the mixture was filtered and the filtrate was dried in vacuum to remove all volatiles. The residue was recrystallized with CH_2_Cl_2_ and gave colourless crystals of the title compound (yield 0.96 g, 74%). ^1^H NMR (300 MHz, CDCl_3_): δ = 7.38–7.25 (m, 4H; phenyl), 3.81 (br, 2H; N*H*), 2.43 (s, 3H; *m*–*MePh*), 2.15–1.21 (m, 11H; *Cy*). ^13^C NMR (75 MHz, CDCl_3_): δ = 139.3–123.6 (*Ph*), 50.4 (*m*–*MePh*), 33.8, 26.5, 25.6, 22.0 (*Cy*). Anal. Calc. for C_14_H_20_N_2_ (216.32): C, 77.73; H, 9.32; N, 12.95%. Found: C, 77.46; H, 9.22; N, 13.04%.

### Refinement

The methyl H atoms were constrained to an ideal geometry, with C—H distances of 0.98Å and *U*_iso_(H) = 1.5*U*_eq_(C), but each group was allowed to rotate freely about its C—C bond. The methylene H atoms were constrained with C—H distances of 0.99Å and *U*_iso_(H) = 1.2*U*_eq_(C). The methine H atom was constrained with C—H distance of 1.00Å and *U*_iso_(H) = 1.2*U*_eq_(C). The phenyl H atoms were placed in geometrically idealized positions and constrained to ride on their parent atoms, with C—H distances in the range 0.95Å and *U*_iso_(H) = 1.2*U*_eq_(C).

The Flack parameter was omitted in CIF because no any atoms heavy Si.

### Crystal data

**Table:**

C_14_H_20_N_2_	*F*(000) = 472
*M**_r_* = 216.32	*D*_x_ = 1.128 Mg m^−^^3^
Orthorhombic, *P*2_1_2_1_2_1_	Mo *K*α radiation, λ = 0.71073 Å
Hall symbol: P 2ac 2ab	Cell parameters from 1347 reflections
*a* = 9.064 (2) Å	θ = 2.8–20.0°
*b* = 11.417 (3) Å	µ = 0.07 mm^−^^1^
*c* = 12.311 (3) Å	*T* = 200 K
*V* = 1274.0 (5) Å^3^	Block, colourless
*Z* = 4	0.30 × 0.25 × 0.20 mm

### Data collection

**Table:**

Bruker SMART CCD diffractometer	2244 independent reflections
Radiation source: fine–focus sealed tube	1758 reflections with *I* > 2σ(*I*)
Graphite monochromator	*R*_int_ = 0.042
φ and ω scan	θ_max_ = 25.0°, θ_min_ = 2.4°
Absorption correction: multi-scan (*SADABS*; Sheldrick, 1996)	*h* = −10→7
*T*_min_ = 0.980, *T*_max_ = 0.987	*k* = −13→12
7147 measured reflections	*l* = −14→14

### Refinement

**Table:**

Refinement on *F*^2^	Secondary atom site location: difference Fourier map
Least-squares matrix: full	Hydrogen site location: inferred from neighbouring sites
*R*[*F*^2^ > 2σ(*F*^2^)] = 0.040	H atoms treated by a mixture of independent and constrained refinement
*wR*(*F*^2^) = 0.098	*w* = 1/[σ^2^(*F*_o_^2^) + (0.0452*P*)^2^ + 0.0788*P*] where *P* = (*F*_o_^2^ + 2*F*_c_^2^)/3
*S* = 1.02	(Δ/σ)_max_ < 0.001
2244 reflections	Δρ_max_ = 0.15 e Å^−^^3^
154 parameters	Δρ_min_ = −0.12 e Å^−^^3^
0 restraints	Extinction correction: *SHELXL97* (Sheldrick, 2008), Fc^*^=kFc[1+0.001xFc^2^λ^3^/sin(2θ)]^-1/4^
Primary atom site location: structure-invariant direct methods	Extinction coefficient: 0.020 (3)

### Special details

**Table:**

Geometry. All s.u.'s (except the s.u. in the dihedral angle between two l.s. planes) are estimated using the full covariance matrix. The cell s.u.'s are taken into account individually in the estimation of s.u.'s in distances, angles and torsion angles; correlations between s.u.'s in cell parameters are only used when they are defined by crystal symmetry. An approximate (isotropic) treatment of cell s.u.'s is used for estimating s.u.'s involving l.s. planes.
Refinement. Refinement of *F*^2^ against ALL reflections. The weighted *R*–factor *wR* and goodness of fit *S* are based on *F*^2^, conventional *R*–factors *R* are based on *F*, with *F* set to zero for negative *F*^2^. The threshold expression of *F*^2^ > σ(*F*^2^) is used only for calculating *R*–factors(gt) *etc*. and is not relevant to the choice of reflections for refinement. *R*–factors based on *F*^2^ are statistically about twice as large as those based on *F*, and *R*–factors based on ALL data will be even larger.

### Fractional atomic coordinates and isotropic or equivalent isotropic displacement parameters (Å^2^)

**Table:**

	*x*	*y*	*z*	*U*_iso_*/*U*_eq_	
N1	0.12298 (18)	0.22773 (14)	0.08238 (13)	0.0434 (4)	
N2	−0.08387 (18)	0.17325 (15)	−0.02421 (15)	0.0475 (5)	
C1	0.1003 (2)	0.14588 (16)	0.17183 (15)	0.0391 (5)	
H1B	−0.0079	0.1422	0.1872	0.047*	
C2	0.1528 (2)	0.02235 (17)	0.14538 (16)	0.0502 (6)	
H2C	0.0991	−0.0073	0.0810	0.060*	
H2D	0.2593	0.0240	0.1277	0.060*	
C3	0.1267 (3)	−0.05956 (17)	0.24111 (18)	0.0611 (7)	
H3A	0.0195	−0.0656	0.2553	0.073*	
H3B	0.1638	−0.1387	0.2228	0.073*	
C4	0.2041 (3)	−0.0156 (2)	0.34235 (18)	0.0612 (7)	
H4A	0.3122	−0.0170	0.3308	0.073*	
H4B	0.1809	−0.0679	0.4042	0.073*	
C5	0.1552 (3)	0.10824 (19)	0.36872 (17)	0.0612 (6)	
H5A	0.2124	0.1376	0.4316	0.073*	
H5B	0.0496	0.1075	0.3894	0.073*	
C6	0.1769 (2)	0.19043 (17)	0.27290 (16)	0.0485 (5)	
H6A	0.1375	0.2687	0.2916	0.058*	
H6B	0.2837	0.1989	0.2581	0.058*	
C7	0.0332 (2)	0.23343 (16)	−0.00570 (16)	0.0382 (5)	
C8	0.0761 (2)	0.32275 (16)	−0.08846 (16)	0.0387 (5)	
C9	0.1108 (2)	0.43699 (16)	−0.05829 (17)	0.0460 (5)	
H9A	0.1104	0.4587	0.0162	0.055*	
C10	0.1457 (3)	0.51857 (19)	−0.13683 (18)	0.0559 (6)	
H10A	0.1683	0.5967	−0.1162	0.067*	
C11	0.1480 (3)	0.48753 (19)	−0.24477 (19)	0.0578 (6)	
H11A	0.1724	0.5446	−0.2980	0.069*	
C12	0.1153 (2)	0.37453 (19)	−0.27700 (17)	0.0520 (6)	
C13	0.0782 (2)	0.29362 (17)	−0.19718 (16)	0.0446 (5)	
H13A	0.0536	0.2160	−0.2181	0.054*	
C14	0.1184 (3)	0.3393 (2)	−0.3945 (2)	0.0864 (9)	
H14A	0.0928	0.2562	−0.4010	0.130*	
H14B	0.2174	0.3523	−0.4240	0.130*	
H14C	0.0469	0.3864	−0.4352	0.130*	
H2A	−0.098 (2)	0.1201 (17)	0.0303 (17)	0.054 (6)*	
H1A	0.213 (2)	0.2603 (17)	0.0752 (16)	0.052 (6)*	

### Atomic displacement parameters (Å^2^)

**Table:**

	*U*^11^	*U*^22^	*U*^33^	*U*^12^	*U*^13^	*U*^23^
N1	0.0400 (10)	0.0501 (10)	0.0401 (9)	−0.0084 (9)	−0.0028 (8)	0.0139 (8)
N2	0.0419 (10)	0.0512 (10)	0.0495 (11)	−0.0071 (9)	−0.0050 (8)	0.0109 (9)
C1	0.0377 (10)	0.0413 (11)	0.0384 (11)	−0.0025 (9)	0.0019 (9)	0.0065 (8)
C2	0.0573 (14)	0.0481 (12)	0.0451 (13)	0.0004 (11)	−0.0039 (11)	−0.0022 (10)
C3	0.0705 (17)	0.0405 (11)	0.0721 (16)	−0.0032 (12)	−0.0081 (14)	0.0079 (12)
C4	0.0729 (16)	0.0571 (14)	0.0536 (15)	0.0025 (14)	−0.0025 (13)	0.0189 (11)
C5	0.0785 (16)	0.0640 (15)	0.0410 (13)	0.0052 (13)	−0.0004 (12)	0.0068 (11)
C6	0.0565 (13)	0.0438 (11)	0.0450 (12)	0.0025 (11)	−0.0040 (10)	−0.0009 (10)
C7	0.0364 (10)	0.0379 (11)	0.0402 (12)	0.0025 (9)	0.0009 (9)	0.0016 (9)
C8	0.0342 (10)	0.0392 (11)	0.0427 (12)	0.0062 (9)	−0.0006 (9)	0.0042 (9)
C9	0.0502 (13)	0.0430 (11)	0.0448 (12)	0.0024 (10)	−0.0028 (10)	0.0033 (10)
C10	0.0701 (16)	0.0387 (11)	0.0590 (15)	−0.0026 (11)	−0.0040 (12)	0.0082 (11)
C11	0.0638 (16)	0.0524 (13)	0.0570 (15)	−0.0022 (12)	0.0016 (12)	0.0212 (12)
C12	0.0552 (14)	0.0590 (13)	0.0418 (13)	0.0052 (12)	0.0000 (11)	0.0089 (11)
C13	0.0460 (12)	0.0448 (11)	0.0431 (12)	0.0012 (10)	−0.0011 (9)	0.0006 (10)
C14	0.123 (2)	0.0921 (19)	0.0440 (14)	−0.0059 (19)	0.0068 (16)	0.0102 (14)

### Geometric parameters (Å, º)

**Table:**

N1—C7	1.357 (2)	C5—H5B	0.9900
N1—C1	1.459 (2)	C6—H6A	0.9900
N1—H1A	0.90 (2)	C6—H6B	0.9900
N2—C7	1.284 (2)	C7—C8	1.493 (3)
N2—H2A	0.91 (2)	C8—C13	1.379 (3)
C1—C6	1.513 (3)	C8—C9	1.392 (3)
C1—C2	1.524 (3)	C9—C10	1.379 (3)
C1—H1B	1.0000	C9—H9A	0.9500
C2—C3	1.523 (3)	C10—C11	1.375 (3)
C2—H2C	0.9900	C10—H10A	0.9500
C2—H2D	0.9900	C11—C12	1.382 (3)
C3—C4	1.516 (3)	C11—H11A	0.9500
C3—H3A	0.9900	C12—C13	1.390 (3)
C3—H3B	0.9900	C12—C14	1.502 (3)
C4—C5	1.517 (3)	C13—H13A	0.9500
C4—H4A	0.9900	C14—H14A	0.9800
C4—H4B	0.9900	C14—H14B	0.9800
C5—C6	1.520 (3)	C14—H14C	0.9800
C5—H5A	0.9900		
			
C7—N1—C1	123.32 (16)	C1—C6—C5	111.80 (17)
C7—N1—H1A	116.5 (13)	C1—C6—H6A	109.3
C1—N1—H1A	117.7 (13)	C5—C6—H6A	109.3
C7—N2—H2A	110.1 (13)	C1—C6—H6B	109.3
N1—C1—C6	109.93 (15)	C5—C6—H6B	109.3
N1—C1—C2	112.82 (15)	H6A—C6—H6B	107.9
C6—C1—C2	110.11 (16)	N2—C7—N1	127.70 (18)
N1—C1—H1B	107.9	N2—C7—C8	117.35 (18)
C6—C1—H1B	107.9	N1—C7—C8	114.94 (16)
C2—C1—H1B	107.9	C13—C8—C9	118.79 (18)
C3—C2—C1	110.77 (16)	C13—C8—C7	120.08 (17)
C3—C2—H2C	109.5	C9—C8—C7	121.12 (18)
C1—C2—H2C	109.5	C10—C9—C8	119.8 (2)
C3—C2—H2D	109.5	C10—C9—H9A	120.1
C1—C2—H2D	109.5	C8—C9—H9A	120.1
H2C—C2—H2D	108.1	C11—C10—C9	120.4 (2)
C4—C3—C2	111.18 (18)	C11—C10—H10A	119.8
C4—C3—H3A	109.4	C9—C10—H10A	119.8
C2—C3—H3A	109.4	C10—C11—C12	121.0 (2)
C4—C3—H3B	109.4	C10—C11—H11A	119.5
C2—C3—H3B	109.4	C12—C11—H11A	119.5
H3A—C3—H3B	108.0	C11—C12—C13	118.0 (2)
C3—C4—C5	110.44 (19)	C11—C12—C14	121.5 (2)
C3—C4—H4A	109.6	C13—C12—C14	120.5 (2)
C5—C4—H4A	109.6	C8—C13—C12	121.95 (19)
C3—C4—H4B	109.6	C8—C13—H13A	119.0
C5—C4—H4B	109.6	C12—C13—H13A	119.0
H4A—C4—H4B	108.1	C12—C14—H14A	109.5
C4—C5—C6	111.80 (18)	C12—C14—H14B	109.5
C4—C5—H5A	109.3	H14A—C14—H14B	109.5
C6—C5—H5A	109.3	C12—C14—H14C	109.5
C4—C5—H5B	109.3	H14A—C14—H14C	109.5
C6—C5—H5B	109.3	H14B—C14—H14C	109.5
H5A—C5—H5B	107.9		

### Hydrogen-bond geometry (Å, º)

**Table:**

*D*—H···*A*	*D*—H	H···*A*	*D*···*A*	*D*—H···*A*
N1—H1*A*···N2^i^	0.90 (2)	2.08 (2)	2.975 (2)	168.0 (18)

Symmetry code: (i) *x*+1/2, −*y*+1/2, −*z*.
